# Adaptive Covariance Matrix for UAV-Based Visual–Inertial Navigation Systems Using Gaussian Formulas

**DOI:** 10.3390/s25154745

**Published:** 2025-08-01

**Authors:** Yangzi Cong, Wenbin Su, Nan Jiang, Wenpeng Zong, Long Li, Yan Xu, Tianhe Xu, Paipai Wu

**Affiliations:** 1 Institute of Space Sciences, Shandong University, Weihai 264209, China; yzcong@sdu.edu.cn (Y.C.); 202121038@mail.sdu.edu.cn (L.L.); yxu@sdu.edu.cn (Y.X.); thxu@sdu.edu.cn (T.X.); 202217768@mail.sdu.edu.cn (P.W.); 2 China Research Institute of Radio Wave Propagation, Qingdao 266107, China; 19862517628@163.com; 3 Xi’an Research Institute of Surveying and Mapping, Xi’an 710054, China; la9881275@163.com

**Keywords:** drones/UAV, motion blur, image quality assessment, visual–inertial navigation system, adaptive covariance matrix

## Abstract

In a variety of UAV applications, visual–inertial navigation systems (VINSs) play a crucial role in providing accurate positioning and navigation solutions. However, traditional VINS struggle to adapt flexibly to varying environmental conditions due to fixed covariance matrix settings. This limitation becomes especially acute during high-speed drone operations, where motion blur and fluctuating image clarity can significantly compromise navigation accuracy and system robustness. To address these issues, we propose an innovative adaptive covariance matrix estimation method for UAV-based VINS using Gaussian formulas. Our approach enhances the accuracy and robustness of the navigation system by dynamically adjusting the covariance matrix according to the quality of the images. Leveraging the advanced Laplacian operator, detailed assessments of image blur are performed, thereby achieving precise perception of image quality. Based on these assessments, a novel mechanism is introduced for dynamically adjusting the visual covariance matrix using a Gaussian model according to the clarity of images in the current environment. Extensive simulation experiments across the EuRoC and TUM VI datasets, as well as the field tests, have validated our method, demonstrating significant improvements in navigation accuracy of drones in scenarios with motion blur. Our algorithm has shown significantly higher accuracy compared to the famous VINS-Mono framework, outperforming it by 18.18% on average, as well as the optimization rate of RMS, which reaches 65.66% for the F1 dataset and 41.74% for F2 in the field tests outdoors.

## 1. Introduction

Unmanned Aerial Vehicles (UAVs) have attracted significant interest in recent years owing to their vast potential across diverse domains such as autonomous navigation [1,2,3], surveillance [4,5,6], and environmental monitoring [7,8]. Visual–Inertial Navigation Systems (VINS) are critical to these applications, providing accurate and reliable localization and mapping capabilities [9,10,11,12]. By integrating VINS, UAVs achieve precise navigation and operational functionality in complex environments, enabling missions including search and rescue [13,14], agricultural monitoring [15], and infrastructure inspection [16]. These systems accomplish Simultaneous Localization and Mapping (SLAM) through sensor data fusion, empowering UAVs to autonomously determine their position and construct accurate maps of unknown surroundings. The integration of visual and inertial sensors provides robust and precise localization, essential for autonomous navigation in dynamic settings for drones. This fusion enhances localization accuracy and robustness, overcoming the limitations of using either sensor alone. The significance of VINS in UAV applications lies in the (1) ability to deliver high precision in pose estimation, which is critical for effective drone navigation; (2) enhanced robustness against environmental changes, making it suitable for both indoor and outdoor use; and (3) cost-effectiveness and compactness, as cameras and IMUs are relatively inexpensive and lightweight. Despite its advantages, VINS faces several challenges that limit its performance in real-world drone applications.

In UAV-based VINS, image quality can significantly vary due to factors such as lighting conditions [17,18], motion blur [19], and environmental dynamics [20,21], which can profoundly impact the accuracy of feature extraction and tracking [22]. UAVs often operate in challenging environments with rapidly changing scenes, and variations in lighting, such as transitions between indoor and outdoor settings, or in high-dynamic range scenes, can create substantial difficulties for visual–inertial systems. In low-light conditions, images captured by UAVs may suffer from high noise levels [17], which can reduce the reliability of feature detection. Furthermore, UAVs operating at high speeds or making sharp maneuvers are particularly prone to motion blur, where rapid movement of the camera or objects within the scene can cause smeared features, making accurate tracking difficult [23,24]. These variations in image quality can result in degraded localization performance and unstable system behavior, which is a critical concern for reliable UAV navigation [3]. Addressing these challenges requires sophisticated image processing techniques that can assess and adapt to changes in image quality, particularly for UAVs. For example, advanced blur detection algorithms and adaptive filtering methods can be employed to maintain feature tracking reliability under varying environmental conditions [25,26].

However, existing methods generally overlook the impact of image quality, particularly motion blur, on navigation accuracy. Motion blur, caused by the rapid movement of UAVs, results in displacement during the imaging process, causing objects to appear smeared [19]. This blur not only reduces image quality but also complicates feature point extraction, increasing the uncertainty of feature points and leading to errors in feature matching and position estimation [20,22,27]. Traditional methods often employ fixed visual covariance matrix settings, which are not adaptable to variations in image quality, thereby restricting the system’s ability to flexibly adjust to dynamic UAVs with fluctuating visual conditions [10,11,28,29]. For another, the covariance matrix is pivotal in ensuring accurate and reliable state estimation by quantifying and propagating uncertainties within VINS, particularly in UAV applications [2,30,31,32]. This matrix, essential in statistical analysis and state estimation, represents the uncertainty associated with various state estimates, such as positions, velocities, orientations, and sensor biases [27,33,34]. Its primary function is to reflect the degree to which two variables change together (covariance) and the individual uncertainties (variance) of each state variable. This is particularly important when operating in environments where rapid changes or unpredictable obstacles are encountered. The co-variance matrix serves essential roles in UAV navigation systems:

Sensor Fusion [30,31,34,35]: The covariance matrix is instrumental in optimally combining measurements from the camera and IMU. This fusion improves the robustness and accuracy of state estimates, enabling UAVs to perform reliably in complex and visually challenging environments.

State Propagation [36,37]: The covariance matrix is integral to state propagation and updates in the extended Kalman filter (EKF) framework, where it helps predict new states and their associated uncertainties. This allows UAVs to adjust and maintain accurate estimations of their position and velocity as they move through changing environments.

Pose Graph Optimization [31,38,39]: For UAVs navigating over large, complex areas, the covariance matrix is crucial during loop closure and Global Pose Graph Optimization. It helps integrate loop closure constraints, accounting for uncertainties and improving the accuracy of the global map, ensuring that UAVs can perform autonomous operations over extended periods and across large, unfamiliar spaces.

Furthermore, the covariance matrix plays a critical role during IMU pre-integration, propagating measurement uncertainties to aid state initialization in visual–inertial bundle adjustment [10,40,41]. Within sliding window optimization, it balances sensor contributions by weighting measurements according to their uncertainty and recency. By monitoring the evolution of the covariance matrix, VINS can detect potential failures in the estimation process, enabling timely corrective actions to maintain robustness [3]. Thus, the covariance matrix is a fundamental component in VINS, enabling it to achieve high accuracy and robustness, making it suitable for applications such as autonomous UAV navigation and augmented reality [32,33,42].

Therefore, this study proposes an innovative adaptive covariance matrix framework for UAV-based VINS to address limitations arising from variable image quality. Our core research objective is to enhance VINS’s performance in drone applications through dynamic covariance adjustment based on real-time image blur assessment. Specifically, we leverage the Laplacian operator—an advanced image processing technique—to quantify blur levels critical for UAVs operating in diverse environments. These assessments enable dynamic adaptation of the visual covariance matrix, optimizing measurement weighting according to instantaneous image clarity with Gaussian formulas. The main contributions can be summarized as follows:

- A novel covariance matrix estimation method is proposed to efficiently adapt to the image quality, where the Laplacian operator is utilized to evaluate the motion blur score.
- A novel VINS framework is constructed, transforming the adaptive covariance matrix into visual uncertainties using Gaussian formulas to improve the system’s performance, especially in dynamic environments.
- Extensive simulation and field experiments validate the effectiveness of our method, demonstrating significant improvements in navigation performance compared to the traditional VINS method.

This approach seeks to improve navigation accuracy and robustness for drones, especially in scenarios involving motion blur that may arise during high-speed flight or maneuvering. The expected effects of this study include enhanced localization accuracy and system robustness in dynamic environments, providing a practical solution to the challenges posed by varying image quality in VINS applications for UAVs.

## 2. Methodology

### 2.1. System Overview

**Figure 1:**
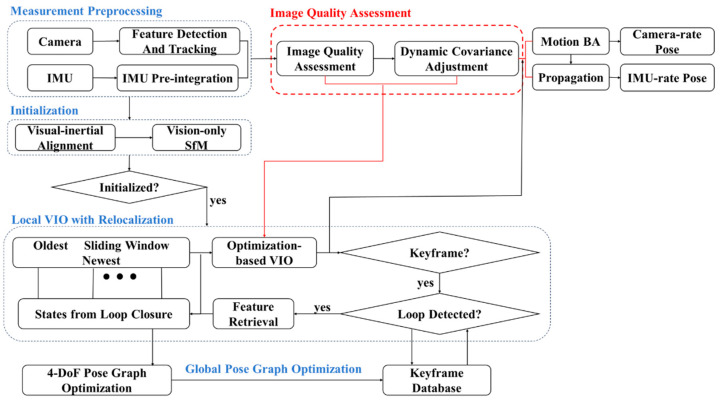
Adaptive visual–inertial navigation architecture based on image quality judgment, where red marks the main contributions.

As illustrated by Figure 1, the architecture of the proposed algorithm consists of five main modules: Measurement Preprocessing, Initialization, Local VIO with Relocalization, Image Quality Assessment, and Global Pose Graph Optimization.

Measurement Preprocessing: This module handles the initial collection and preprocessing of data from the camera and IMU sensors. The raw data inputs include images captured by the camera, angular velocities measured by the gyroscope, and acceleration values from the accelerometer. The system utilizes the Shi–Tomasi corner detection method [43] to identify salient feature points within the images. This method is preferred due to its robustness and efficiency in detecting corners that are suitable for tracking. The Shi–Tomasi method calculates the minimum eigenvalue of the gradient matrices, selecting points with a high response, which indicates strong corners. For tracking the detected feature points, we employ the Kanade–Lucas–Tomasi (KLT) tracker [43]. The KLT tracker is effective for maintaining correspondences of feature points across consecutive frames. It uses an iterative approach to minimize the displacement error between feature points in the previous frame and their projected positions in the current frame. This method is advantageous for its computational efficiency and accuracy in tracking small motions.

Image Quality Assessment: Image quality can significantly vary due to lighting conditions, motion blur, and environmental factors, impacting the accuracy of feature extraction and tracking. To address this, we employ the Laplacian operator to assess image quality by quantifying the degree of blur in an image. The degree of blur is determined by measuring the variance or standard deviation of the Laplacian result, with higher variance indicating a sharper image and lower variance indicating a blurrier image. The covariance matrix is dynamically adjusted based on this assessment to reflect the current image quality. In the subsequent optimization-based VIO (visual–inertial-odometry) step, the system uses a sliding window approach to optimize states, leveraging the dynamically adjusted covariance matrix to adaptively weight visual and inertial measurements. This is followed by motion bundle adjustment (BA), where the adjusted covariance matrix enhances the robustness of the trajectory optimization.

Initialization: This step estimates essential initial states such as the gravity vector, velocity, gyroscope bias, and metric scale. This initialization ensures that the system starts with high-quality initial states, which is crucial for robust performance. The initialization leverages visual measurements to perform structure from motion (SfM) for a short period, determining three-dimensional coordinates and camera poses using methods like efficient perspective-n-point (EPnP).

Local VIO with Relocalization: This module utilizes a sliding window approach for nonlinear optimization, constructing a factor graph model. The model consists of nodes representing state variables and edges representing measurement constraints (e.g., visual reprojection factors, IMU pre-integration factors as detailed in [10]). The sliding window includes the current image frame and previously identified keyframes, which are continually optimized to minimize the overall error. The dynamically adjusted covariance matrix from the image quality assessment step is used to adaptively weigh the visual and inertial measurements, enhancing the robustness of the state estimates.

Global Pose Graph Optimization: Loop closure is essential for correcting drift over long trajectories. The system employs a loop detection mechanism to identify previously visited locations [44]. When a loop is detected, loop closure constraints are integrated into the optimization process using the covariance matrix to account for uncertainties. This helps refine the global pose estimates and improve the overall map consistency. The Global Pose Graph Optimization adjusts the global trajectory using loop closure constraints stored in the keyframe database. This structured process ensures that the system remains accurate and robust across various environmental conditions by continuously adapting to image quality variations.

In the following, the main contributions of our proposed method will be illustrated literally, while the details for other procedures can be found in [10] on account of the length of the paper.

### 2.2. Image Quality Calculation

Image blur is a critical parameter in image processing that measures the clarity or sharpness of an image. The Laplacian algorithm is a popular method used to quantify image blur due to its sensitivity to edges. This section provides a detailed, scientifically rigorous description of the Laplacian algorithm, including the relevant mathematical formulations and a flowchart illustrating the algorithmic steps.

The Laplacian of an image is a second-order derivative that highlights regions of rapid intensity change, such as edges. The mathematical basis of the Laplacian operator in two dimensions $I_{i}\left(x,y\right)$ is given by the following discrete approximation:

(1) $$L_{i}=Laplacian(I_{i})=\frac{\partial^{2}I_{i}}{\partial x^{2}}+\frac{\partial^{2}I_{i}}{\partial y^{2}}$$

where $I_{i}$ is the pixel value. In digital image processing, the discrete Laplacian operator is typically represented using a convolution kernel. One of the commonly used kernels is

(2) $$\mathrm{Laplacian}\;\mathrm{Kernel}\;=\left[\begin{array}{ccc} 0 & -1 & 0\\ -1 & 4 & -1\\ 0 & -1 & 0 \end{array}\right]$$

The degree of blur in an image can then be quantified by measuring the variance or standard deviation of the Laplacian result. A higher variance indicates a sharper image, while a lower variance indicates a blurrier image. The variance is calculated as follows:

(3) $$\sigma^{2}=\frac{1}{N}\sum_{i=1}^{N}{\left(L_{i}-\mu \right)}^{2}$$

where N is the number of pixels, $L_{i}$ is the Laplacian value at the i-th pixel, and μ is the mean Laplacian value.

As in Algorithm 1, the following steps outline the overall process of calculating image blur using the Laplacian algorithm:

(a) Image Acquisition: Obtain the image to be analyzed (Line 2–3).
(b) Gaussian Smoothing: Apply Gaussian smoothing to the image to reduce noise (Line 4–7).
(c) Laplacian Calculation: Compute the Laplacian of the smoothed image using the convolution kernel (Line 8–9).
(d) Variance Computation: Calculate the variance of the Laplacian result to quantify the image blur (Line 10–17).

This approach provides a robust measure of image quality, which is then used to dynamically adjust the covariance matrix for VINS.
**Algorithm 1: Calculate Image Blur Using Laplacian Algorithm****Input:** Image
**Output:** Image Blur
1: function CALCULATE_IMAGE_BLUR(image)
2:           image ← LOAD_IMAGE(image)
3:           (h, w) ← DIMENSIONS(image)
4:           sigma ← 1.0
5:           gaussian_kernel_size ← 3
6:           gaussian_kernel ←GAUSSIAN_KERNEL(gaussian_kernel_size, sigma)
7:           smoothed_image ← CONVOLVE(image, gaussian_kernel, (h, w))
8:           laplacian_kernel ← [[0, −1, 0], [−1, 4, −1], [0, −1, 0]]
9:           laplacian_image ← CONVOLVE(smoothed_image, laplacian_kernel, (h, w))
10:         sum_pixels ← 0
11:         for each pixel in laplacian_image do
12:              sum_pixels ← sum_pixels + pixel
13:         mean ← sum_pixels/(h * w)
14:         variance_sum ← 0
15:         for each pixel in laplacian_image do
16:              variance_sum ← variance_sum + (pixel − mean)^2
17:         Image Blur ← variance_sum/(h * w)
18:         return Image Blur
19: end function

### 2.3. Adaptive Covariance Matrix Estimation

In traditional VINS, the visual covariance matrix remains fixed, limiting the system’s adaptability to varying image conditions, such as motion blur. The proposed method introduces a dynamic adjustment mechanism for the covariance matrix based on real-time image quality assessment, specifically targeting the impacts of motion blur on the navigation system. To dynamically adjust the covariance matrix, the system first assesses the image quality using the Laplacian operator. The sharpness or blurriness of an image can be quantified by measuring the variance of the Laplacian response. Higher variance indicates a sharper image, while lower variance indicates a blurrier image, as illustrated in Section 2.1. Based on this assessment, the system dynamically adjusts the visual covariance matrix, proportionally scaling it to the calculated variance. When the variance is low (indicating blur), the covariance values are increased to reflect higher uncertainty. Conversely, when the variance is high (indicating a sharp image), the covariance values are decreased to reflect lower uncertainty. To ensure a scientifically sound adjustment mechanism, we conducted extensive statistical analysis on a large dataset of images to assess their quality distribution. We performed a Gaussian fit on the image quality data, confirming that the distribution adhered to a Gaussian model. Based on this fit, we developed a function where image quality serves as the independent variable, and the covariance matrix coefficient is the dependent variable. This function integrates a penalty and reward mechanism into the covariance matrix, dynamically adjusting the weights according to the quality of the images. The function is expressed as follows:

(4) $$f(x)=ae^{-\frac{{\left(x-b\right)}^{2}}{2c^{2}}}$$

where f(x) represents the covariance matrix coefficient as a function of image quality, a is the amplitude coefficient, which determines the peak height of the Gaussian curve, e is the base of the natural logarithm, approximately equal to 2.71828, x is the independent variable, representing image quality, b is the mean, indicating the central position of the Gaussian curve, and c is the standard deviation, indicating the width of the curve and the degree of dispersion. A larger c value results in a wider and flatter curve. This Gaussian function enables the system to dynamically adjust the covariance matrix based on real-time image quality assessments, thereby enhancing the robustness and accuracy of the VINS by appropriately weighting images of varying quality.

### 2.4. Adaptive Integration of Visual–Inertial Odometry with Global Optimization

**Figure 2:**
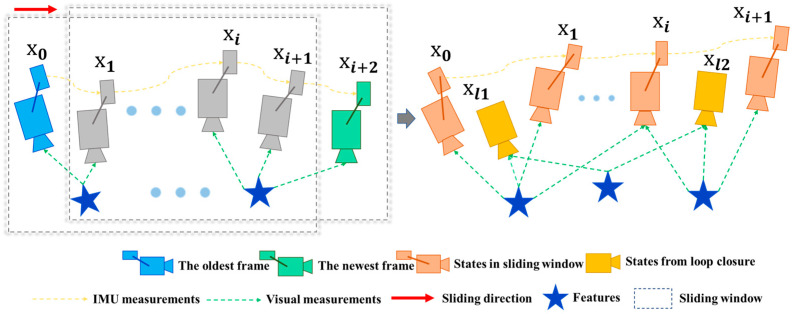
Illustration of the sliding window monocular VIO with re-localization.

To further enhance the performance of VINS, the integration of VIO with global optimization techniques is critical. This section describes how global optimization is incorporated into the VINS framework to improve the overall system accuracy and robustness. As shown in Figure 2, the optimization process of our system comprises local sliding window optimization and Global Pose Graph Optimization. In this process, we formulate a nonlinear optimization problem by integrating visual feature re-projection errors and IMU pre-integration errors. The optimization variables include the poses and velocities of all frames within the sliding window, as well as the 3D positions of feature points. During sliding window optimization, to keep computational complexity manageable, we remove the oldest frame within the sliding window while retaining its critical information for the current optimization problem. This is achieved through marginalization, which transfers the constraints of the removed frame to the remaining frames. We then employ nonlinear optimization algorithms, such as the Levenberg–Marquardt method, to minimize the re-projection and IMU pre-integration errors, thereby optimizing the poses and velocities of all frames within the sliding window.

This paper introduces an image quality perception function to construct an adaptive observation covariance matrix that dynamically correlates with the reliability of image observations. This matrix is employed to adjust the weight of observation terms during graph optimization. The proposed method leverages image quality assessment to adaptively modulate the weighting coefficients of each observation frame in the objective function, thereby achieving a more robust optimization outcome. In the objective function that minimizes visual residuals, the system refines camera pose estimation by minimizing the reprojection error. To further characterize the statistical properties of visual observation errors, this work models them as random variables following a two-dimensional Gaussian distribution. The visual observation errors are typically assumed to have zero mean and a fixed covariance. The error term is modeled as follows:

(5) $$e_{i}=p_{\mathrm{proj},i}-p_{\mathrm{obs},i}\sim N\left(0,\sigma^{2}I_{2}\right)$$

where $p_{\mathrm{proj},i}$ denotes the projected position of the feature point in the current frame and represents the actual observed position in the image.

This paper introduces an image quality-aware mechanism that dynamically adjusts the covariance of observation errors through a perceptual function. The covariance is extended into a form that incorporates image quality awareness and is modeled as follows:

(6) $$\Sigma_{i}=f{\left(x_{i}\right)}^{2}\cdot I_{2}$$

where $x_{i}$ denotes the image quality metric of the i-th observation frame, and $f\left(x_{i}\right)$ represents the covariance adjustment factor based on image quality. The lower the image quality, the larger the covariance of the observation error, indicating a lower degree of confidence assigned to the corresponding observation by the system. By incorporating image quality weighting, the observation error covariance matrix $\Sigma_{i}$ can be further inverted to obtain the corresponding information matrix $\Omega_{i}$, expressed as follows:

(7) $$\Omega_{i}=\Sigma_{i}^{-1}=\frac{1}{f{\left(x_{i}\right)}^{2}}\cdot I_{2}$$

In the nonlinear optimization process, the system estimates the state variables by minimizing the weighted sum of squared observation residuals. The residual term is formulated as follows:

(8) $$r_{i}=\sqrt{\Omega_{i}}\cdot \left(p_{\mathrm{proj},i}-p_{\mathrm{obs},i}\right)=\frac{1}{f\left(x_{i}\right)}\cdot \left(p_{\mathrm{proj},i}-p_{\mathrm{obs},i}\right)$$

From the above expression, the weighted optimization objective function for the visual observations can be formulated as

(9) $$\min\sum_{i}{\left\|r_{i}\right\|}^{2}=\sum_{i}\frac{1}{f{\left(x_{i}\right)}^{2}}\cdot {\left\|p_{\mathrm{proj},i}-p_{\mathrm{obs},i}\right\|}^{2}$$

This weighting mechanism ensures that observations from lower-quality images are assigned reduced weights in the objective function, thereby diminishing their influence on the final state estimation. Conversely, observations from high-quality images are associated with smaller covariances, resulting in amplified residuals. This allows the optimizer to more effectively exploit reliable information for accurate state estimation.

Furthermore, within the sparse nonlinear optimization framework, the contribution of each factor to the system’s overall information matrix is given by the following:

(10) $$H_{i}=J_{i}^{⊤}\cdot \Omega_{i}\cdot J_{i}=\frac{1}{f{\left(x_{i}\right)}^{2}}\cdot J_{i}^{⊤}\cdot J_{i}$$

This implies that high-quality observations contribute stronger second-order information, which facilitates faster convergence and improved estimation accuracy of the optimizer. In contrast, the influence of errors from low-quality images is explicitly attenuated at the structural level, enhancing the robustness of the system.

By incorporating image quality-aware weighting into the visual–inertial joint optimization framework, a fundamental optimization objective function is constructed, integrating both IMU pre-integration errors and visual observation errors:

(11) $$\underset{X}{\min}\left(\sum_{k}{\left\|r_{\mathrm{imu},k}\right\|}^{2}+\sum_{i}\sum_{j\in O(i)}\frac{1}{f{\left(x_{i}\right)}^{2}}\cdot {\left\|p_{\mathrm{proj}}^{ij}-p_{\mathrm{obs}}^{ij}\right\|}^{2}\right)$$

The above optimization objective primarily targets the basic VIO process, where X denotes the set of state variables to be optimized, considering only IMU pre-integration errors $r_{\mathrm{imu},k}$ and visual observation errors. Building upon this, this paper further incorporates prior information into the sliding-window optimization framework to construct a complete visual–inertial optimization model, which is formulated as follows:

(12) $$\underset{X}{\min}\left\{\begin{array}{l} {\left\|r_{p}-H_{p}X\right\|}^{2}+\sum_{k\in B}{\left\|r_{B}\left({\hat{z}}_{b_{k}}^{b_{k+1}},X\right)\right\|}_{P_{b_{k+1}}^{-1}}^{2}\\ +\sum_{(l,j)\in C}\rho \left({\left\|r_{C}\left({\hat{z}}_{l}^{c_{j}},X\right)\right\|}_{{\left(f{\left(x_{j}\right)}^{2}\cdot P_{l}^{c_{j}}\right)}^{-1}}^{2}\right) \end{array}\right\}$$

where $r_{p}$ represents the prior residual; $H_{p}$ is the prior information matrix; $r_{B}\left({\hat{z}}_{b_{k}}^{b_{k+1}},X\right)$ denotes the IMU pre-integration residual; $P_{b_{k+1}}^{-1}$ is the covariance matrix associated with the IMU pre-integration error; $r_{C}$ represents the reprojection error; and $P_{l}^{c_{j}}$ is the covariance matrix corresponding to the visual observation.

During the relocalization process, once a loop closure is detected, loop feature observations are introduced to enhance state constraints. In this work, image quality-aware weighting is incorporated into the loop closure residuals. The revised relocalization optimization objective function is formulated as follows:

(13) $$\underset{X}{\min}\left\{\begin{array}{l} {\left\|r_{p}-H_{p}X\right\|}^{2}+\sum_{k\in B}{\left\|r_{B}\left({\hat{z}}_{b_{k}}^{b_{k+1}},X\right)\right\|}_{P_{b_{k+1}}^{-1}}^{2}\\ +\sum_{(l,j)\in C}\rho \left({\left\|r_{C}\left({\hat{z}}_{l}^{c_{j}},X\right)\right\|}_{{\left(f{\left(x_{j}\right)}^{2}\cdot P_{l}^{c_{j}}\right)}^{-1}}^{2}\right)\\ +\sum_{(l,v)\in L}\rho \left({\left\|r_{C}\left({\hat{z}}_{l}^{v},X,{\hat{q}}_{v}^{w},{\hat{p}}_{v}^{w}\right)\right\|}_{{\left(f{\left(x_{v}\right)}^{2}\cdot P_{l}^{c_{v}}\right)}^{-1}}^{2}\right) \end{array}\right\}$$

where (l,v)∈L denotes the feature point associated with the loop closure constraint and the corresponding loop frame; ${\hat{q}}_{v}^{w}$ and ${\hat{p}}_{v}^{w}$ represent the estimated rotation and translation of the loop frame v in the world coordinate system, respectively; and $P_{l}^{c_{v}}$ denotes the covariance matrix of the loop observation.

By integrating the proposed adaptive covariance matrix with VIO and global optimization through detailed mathematical formulations, VINS achieves a high level of accuracy and robustness in state estimation. This integration allows the system to effectively handle various image quality issues caused by rapid motion or dynamic environments, large-scale mapping, and long-term navigation tasks, making it suitable for large-scale mapping and long-term navigation tasks for a wide range of applications, including autonomous vehicles, drones, and augmented reality systems.

## 3. Experiments and Analysis

### 3.1. Experimental Data

To verify the general navigation applicability of our approach, we evaluated the system using datasets collected with two different hardware setups. The primary datasets are the MH01-05 and V101-203 sequences from the EuRoC dataset [45] and the Room 1–3 sequences from the TUM VI dataset [46].

**Figure 3:**
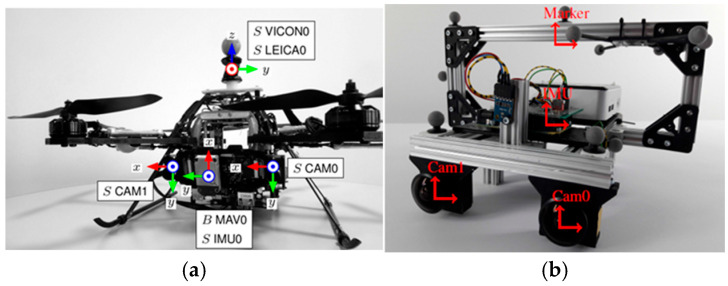
Dataset sampling equipment. (**a**) Euroc. (**b**) TUM VI.

The EuRoC dataset is an open dataset released by the European Conference on Mobile Robots, designed specifically for the evaluation of VINS. This dataset provides a standardized platform for assessing the performance of VINS algorithms in complex and dynamic environments. We focus on two series: the MH series, which captures data in a cluttered machine hall environment, where the Micro Aerial Vehicle (MAV in Figure 3a) flies at high speeds and angular velocities, simulating fast motion and motion blur under low-light conditions; and the V series, which contains sequences recorded in a Vicon Room—a controlled environment that enables more precise assessments of VINS performance under varying conditions.

The TUM VI dataset, developed by the Technical University of Munich, is a comprehensive dataset designed for evaluating VINS algorithms. The Room subset of this dataset, which we used in our experiments, is collected with a handheld stereo camera system (Figure 3b), simulating realistic human motion patterns such as walking, turning, and looking around. This provides a wide range of motion trajectories, enabling thorough testing of VINS algorithms with various motion blur.

**Figure 4:**
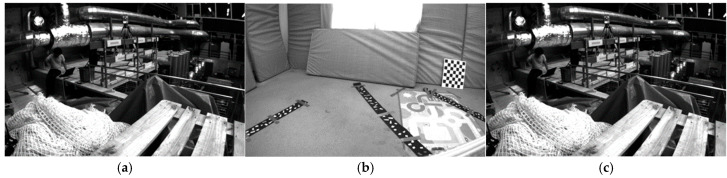
Dataset sampling environment. (**a**) Euroc Room. (**b**) Euroc V. (**c**) TUM VI Room.

Table 1 provides a comprehensive overview of the EuRoC dataset and TUM VI dataset, highlighting the testing environments (Figure 4), distances traveled, and key features of each sequence. This detailed information helps in understanding the diversity and complexity of the dataset, which is essential for evaluating the performance of the system.

### 3.2. Image Blur Processing and Quality Assessment

**Figure 5:**
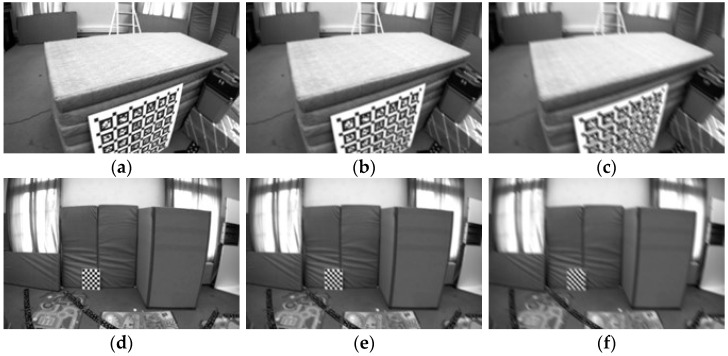
Simulation of motion blur with 45°. (**a**) Original image 1. (**b**) Slow motion blur 1. (**c**) Fast motion blur 1. (**d**) Original image 2. (**e**) Slow motion blur 2. (**f**) Fast motion blur 2.

We initially processed a total of 27,049 images from the dataset to simulate motion blur, utilizing a convolution method. In typical scenarios, horizontal blur is defined as 0°, while vertical blur is defined as 90°. For this study, we selected a 45° directional blur to apply to all images. This specific angle was chosen because it encompasses both horizontal and vertical components, effectively replicating a more comprehensive range of motion blur conditions. Furthermore, using the convolution method, we simulated different degrees of motion blur, including both slow-motion and fast-motion blur. This approach ensures that the simulated blur adequately represents real-world scenarios where motion blur occurs in various directions and at varying speeds, as illustrated below (Figure 5).

**Figure 6:**
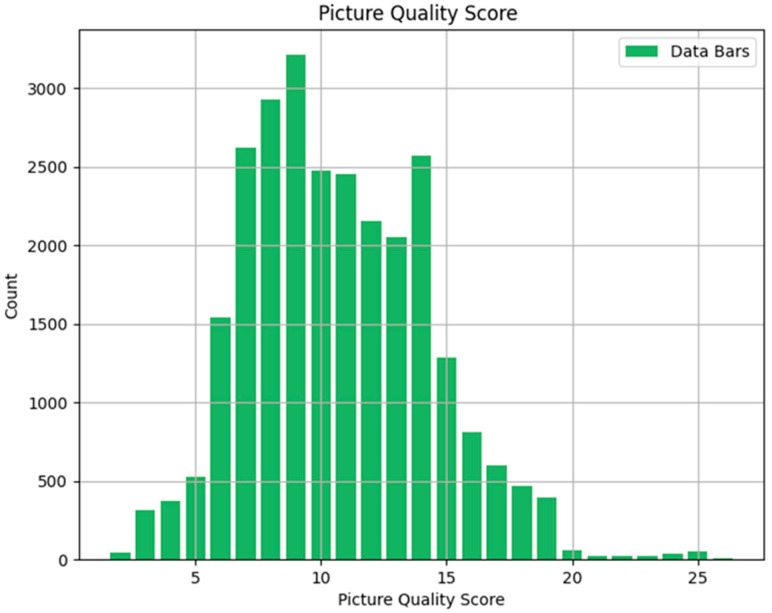
Image quality score statistics.

Subsequently, we evaluated the quality of these blurred images using the Laplacian algorithm, which quantifies image sharpness by measuring the variance of the Laplacian response. We conducted a comprehensive statistical analysis of the image quality scores obtained from this evaluation. Specifically, we performed a frequency analysis of the different image quality scores to understand the distribution and prevalence of various levels of image sharpness across the dataset. The results of this statistical analysis, including the frequency distribution of the image quality scores, are illustrated in Figure 6.

**Figure 7:**
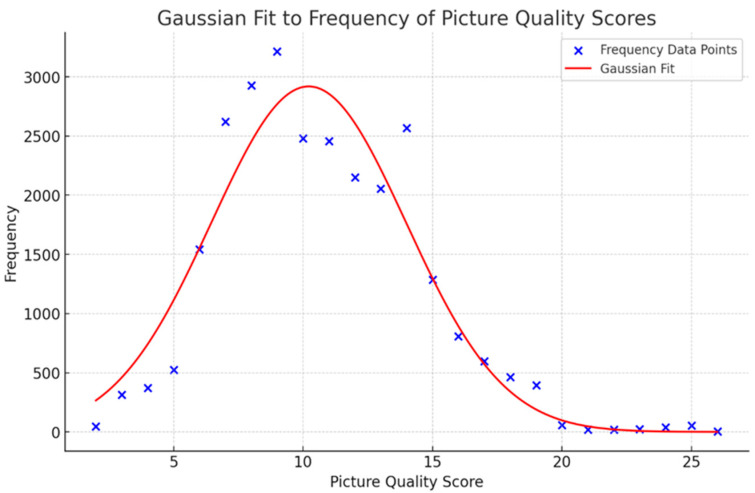
Gaussian fit to frequency of image quality scores.

By observing the frequency distribution of the image quality scores, the majority of the data falls within the range of 6 to 15. Within this interval, the frequency of observations increases as the values approach the center of the range, and decreases as the values move towards the extremes. This trend demonstrates a characteristic Gaussian distribution, where the frequency is highest near the mean and diminishes towards the tails. We noted that the distribution closely follows the characteristics of a normal distribution. Consequently, we applied a Gaussian fitting to the data to model this distribution accurately. This fitting process helps in understanding the underlying statistical properties of the image quality scores and enables us to use these properties for further analysis and adjustments, as illustrated below (Figure 7).

The visual inspection of the fitted curve against the data points indicates a reasonable fit. The Gaussian curve follows the general trend of the data, capturing the peak and spread of the frequency distribution. To quantitatively evaluate the fit, a key metric was calculated: Coefficient of Determination (R^2^): 0.9122. The R^2^ value of 0.9122 indicates that the Gaussian model explains approximately 91.22% of the variance in the data, which is considered a good fit. This high R^2^ value demonstrates that the model effectively captures the main characteristics of the data distribution.

The fitted Gaussian parameters are as follows: amplitude (a): 2918.99, mean (b): 10.22, and standard deviation (c): 3.76. These parameters indicate the peak frequency (amplitude), the central value around which the data is distributed (mean), and the spread of the data (standard deviation).

The Gaussian fit to the image quality score frequency distribution is reasonable based on visual inspection and quantitative analysis. The high R^2^ value suggests that the model captures the main features of the data well, though some discrepancies indicate potential areas for improvement. This analysis demonstrates the effectiveness of the Gaussian model in representing the underlying distribution of the image quality scores.

Based on the degree of blurriness, we adjusted the parameters of the covariance matrix to enhance system robustness. Specifically, we established a function with the image quality score as the independent variable and the covariance matrix parameter as the dependent variable, defined as follows:

(14) $$f\left(x\right)=2\cdot e^{\frac{-{\left(x-1\right)}^{2}}{2\times 10^{2}}}$$

To enhance the system’s accuracy and robustness, we propose an adaptive transformation that modulates the covariance matrix coefficients based on image quality scores. Specifically, we define the (a) as 2, the (b) as 1, and the (c) as 10. This configuration ensures that the maximum value of the covariance matrix coefficients is constrained to 2. As the image quality score improves, the covariance matrix coefficients gradually decrease, eventually stabilizing around 0.1. Conversely, for images of lower quality (lower scores), the coefficients are increased. This adaptive approach significantly improves the precision and resilience of the system by dynamically adjusting the covariance matrix coefficients in response to the quality of the input images. This methodology not only maintains the balance between noise reduction and detail preservation but also provides a scalable solution to varying image quality conditions. The application of such an adaptive covariance matrix proves instrumental in optimizing performance metrics across diverse datasets, thereby substantiating its efficacy and reliability in practical scenarios.

### 3.3. Experimental Analysis of Adaptive VINS System Based on Image Quality

**Figure 8:**
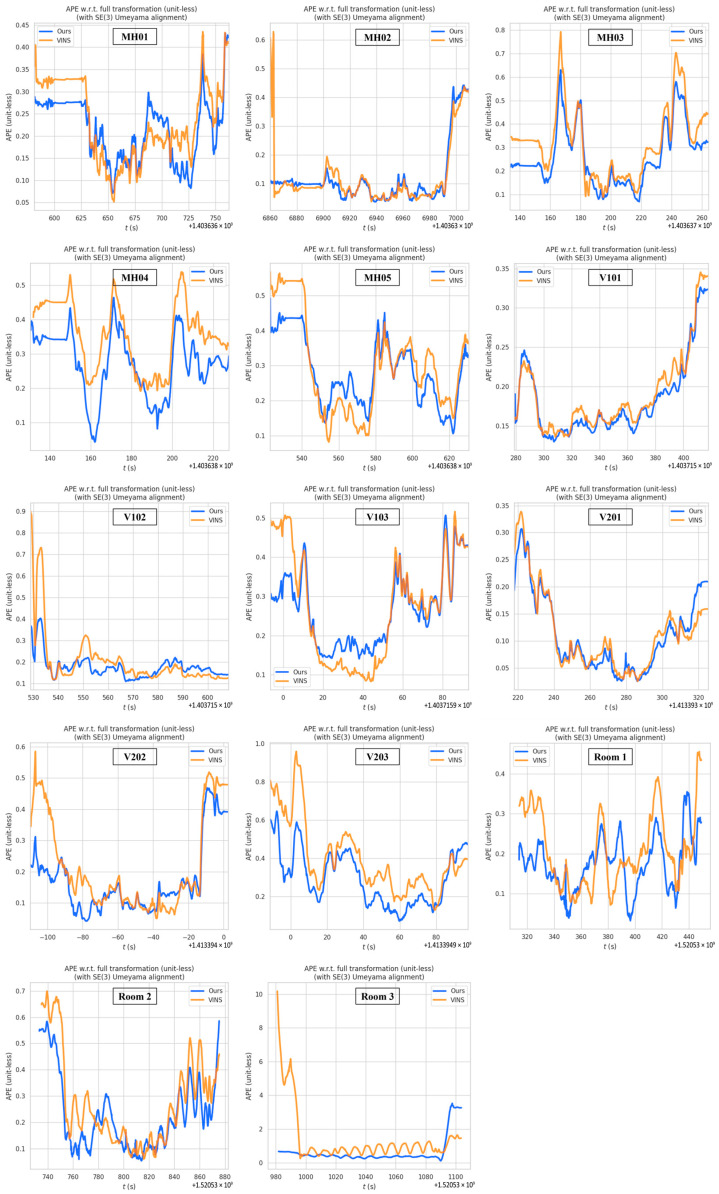
Comparison of absolute pose error with each subfigure corresponding to results of different sequences.

To validate the proposed algorithm, we conducted experiments using the entire EuRoC dataset and TUM VI dataset. The results demonstrated that when the camera experienced high-speed motion, leading to motion blur, the VINS system’s error in feature extraction and optical flow tracking increased. Severe motion blur even caused front-end tracking failures. We compared our results with those of the VINS-Mono (https://github.com/HKUST-Aerial-Robotics/VINS-Mono (accessed on 23 September 2023)) system without loop closure. The absolute pose error (APE) (https://github.com/MichaelGrupp/evo (accessed on 5 March 2024)) for the proposed algorithm was evaluated using the root mean square (RMS) metric on the EuRoC dataset and TUM VI dataset, as shown in Figure 8. The *x*-axis of the figure represents the timestamps from the dataset, specifically the ROS time, which is used to synchronize the sensor data and ensure accurate time alignment for performance evaluation. The *y*-axis represents the RMS of the APE, without unit, providing a clear measurement of the navigation accuracy at full transformation.

Figure 8 illustrates the APE of our system compared to VINS-Mono under conditions of motion blur. The comparative results demonstrate that the proposed algorithm achieves superior positioning accuracy in the initial motion phase compared to VINS-Mono across most of the simulated datasets. The experimental results indicate that under motion blur conditions, feature point detection in images exhibits significant uncertainty, which can easily lead to tracking failures and error accumulation. To address this issue, the proposed algorithm assigns larger covariance values to low-quality image frames, thereby reducing the system’s reliance on such observations and effectively mitigating error accumulation and propagation. The algorithm incorporates a dynamic weight adjustment mechanism into the sliding window optimization process, which adaptively regulates the weights of observation data based on image quality. This approach reduces the risk of system failure caused by unreliable feature points. The adjusted covariance matrix enhances the robustness and convergence stability of the optimization algorithm by assigning lower weights to highly uncertain observations during state estimation, thereby improving overall estimation accuracy. A well-designed covariance matrix also helps decrease the probability of system initialization failure and enhances operational stability under complex motion disturbances. From an overall trend perspective, the absolute pose error curves of the proposed algorithm and VINS-Mono exhibit similar patterns in later motion stages. However, the proposed algorithm maintains a consistently lower error level, demonstrating superior precision performance.

**Figure 9:**
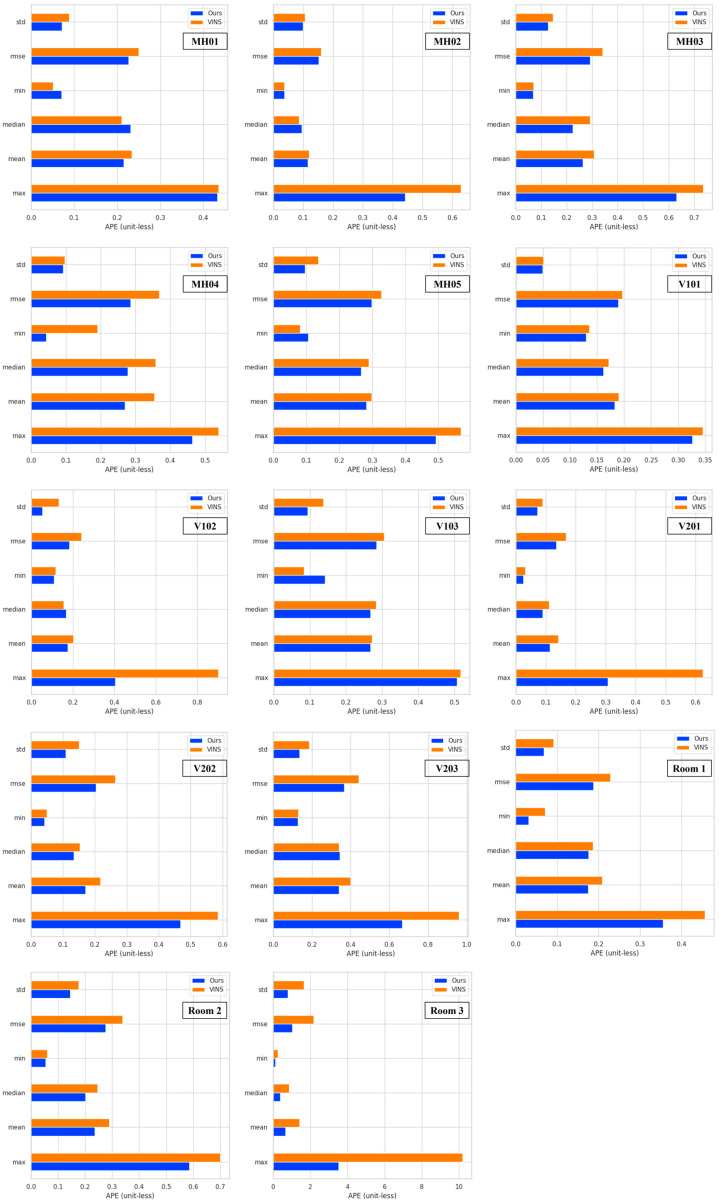
Comparison of accuracy index with each subfigure corresponding to results of different sequences.

However, in the V102 and V201 datasets, the proposed method does not demonstrate a clear advantage, and in some cases, the optimization performance even slightly deteriorates. Analysis of these experimental results reveals that the current system struggles to comprehensively and accurately evaluate all factors affecting image quality. Elements such as image texture and illumination variations may lead to deviations in image quality assessment. Additionally, the Laplacian operator is highly sensitive to noise, potentially misinterpreting image noise as improved sharpness, thereby compromising the accuracy of covariance adjustment. The results are shown in Figure 9.

Figure 9 compares the APE metrics between our system and the VINS-Mono. These metrics include standard deviation (std), root mean square error (rmse), minimum error (min), median error (median), mean error (mean), and maximum error (max). The experimental results demonstrate that the proposed algorithm generally achieves lower error levels in terms of RMSE compared to VINS-Mono. In most simulated datasets, the proposed algorithm shows significant optimization in maximum error, exhibiting stronger robustness and anti-interference capability. However, in the MH01, V101, and V103 simulated datasets, the optimization effect on maximum error is relatively minor and not particularly pronounced. This phenomenon may be attributed to certain limitations of the Laplacian operator in the image quality assessment process, as it cannot fully capture all factors affecting image quality, leading to inaccurate covariance adjustments. If the image quality assessment deviates substantially during covariance adjustment, the optimization algorithm may converge to a local optimum, restricting further reduction in the maximum error. Although optimization effects are less noticeable in individual datasets, the significant improvements in maximum error across other datasets sufficiently demonstrate that the proposed algorithm effectively mitigates the adverse effects of image blur on system performance, highlighting its precision and robustness under extreme conditions. Furthermore, the proposed system generally outperforms the VINS-Mono algorithm in other error metrics, including minimum error and standard deviation. The adaptive covariance adjustment strategy significantly reduces the impact of abnormal observations caused by motion blur, enhancing the overall robustness and positioning accuracy in challenging scenarios. To comprehensively evaluate the precision improvement of the proposed algorithm, the optimization rates of RMSE across all experimental datasets were statistically analyzed. The detailed statistical results are presented in Table 2.

Our algorithm demonstrated significantly higher accuracy compared to the VINS-Mono open framework. Specifically in Table 2, it outperformed VINS-Mono by margins ranging from 3.87% to 52.56%, with an average improvement of 18.18% across the EuRoC dataset and TUM VI dataset. In fact, the different optimization rates are closely related to the key features of the corresponding dataset sequences, with rapid motion and dynamic lighting changes showing the best promotions. The results have shown the adaptability and necessity of our system within complex scenarios.

### 3.4. Field Experiments Outdoors

**Figure 10:**
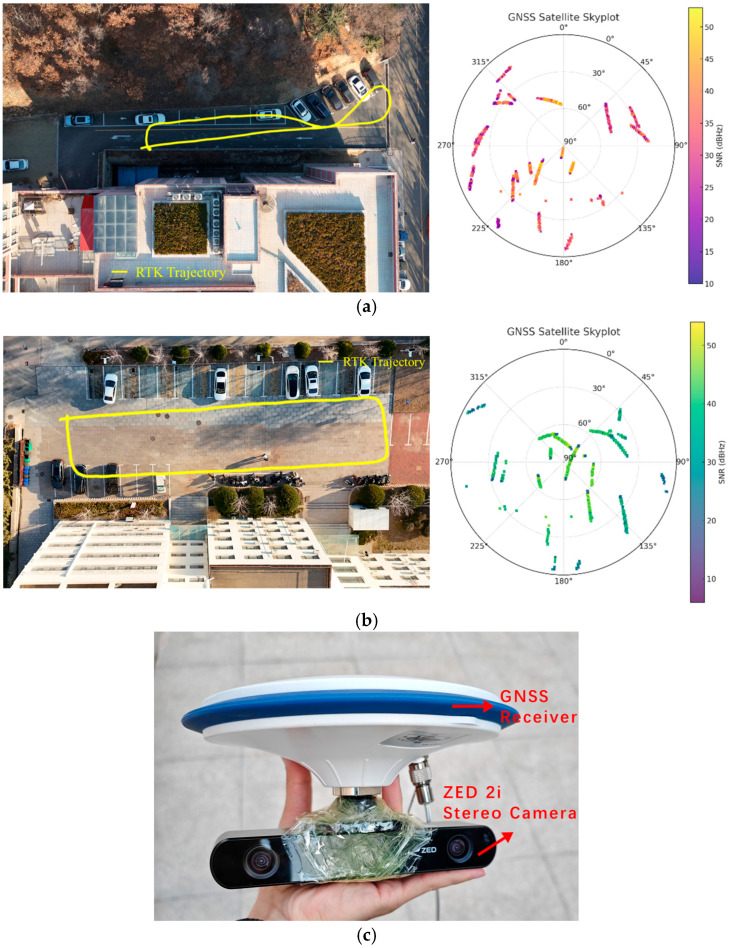
Dataset distributions and equipment specifications. (**a**) F1 dataset. (**b**) F2 dataset. (**c**) hardware setups.

To verify the performance of our method in the field, we collect two sequences of typical datasets using a self-made device with ZED 2i and CYXT N201 GNSS, where RTK is employed as ground truth. As shown in Figure 10, F1 is collected on a narrow slope with about a 10 m height difference, surrounded by trees, cars, and one side of buildings. While the scene in F2 is relatively open, with scattered objects. During the experiments, the equipment is hand-held over the head with people running, simulating the drones flying. The GNSS satellite skyplot for them is illustrated as well. It can be concluded that the elevation angles and SNRs (Signal Noise Ratios) for most satellites are healthy for enough precision of ground truth.

**Figure 11:**
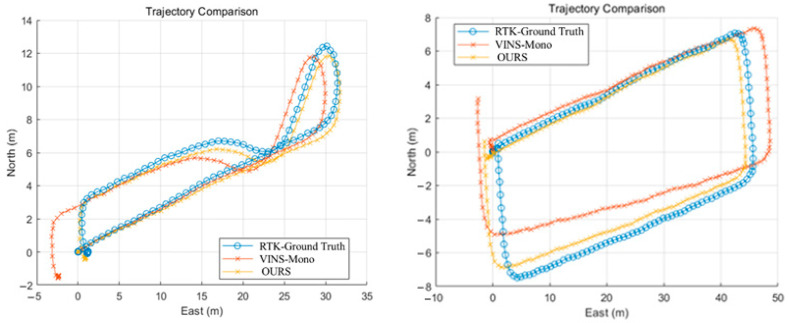
Trajectory comparisons.

Observing Figure 11, it can be seen that the RTK ground truth trajectory is relatively smooth and continuous in the whole closed-loop motion process, which can well reflect the real movement trajectory in both datasets. The trajectory of the traditional VINS-Mono algorithm can closely follow the true value in most sections, and the trajectory trend is basically the same. But, when the steering occurs, there is a slight deviation from the true value of the trajectory, indicating that the algorithm may have a slight cumulative error in the environment of fast attitude change with complex lighting and feature points. In the end, this error gradually accumulates and gradually produces a large offset amplitude with the RTK truth value, and, finally, produces a meter-level error with the truth value. In contrast, the trajectory of the adaptive covariance matrix algorithm, our method, is closer to the RTK truth value as a whole. In particular, the deviation amplitude is smaller during the path corner, resulting in less deviation at the end of the trajectory. It shows that our method has stronger robustness and accuracy maintenance ability in dealing with fast steering or sparse feature environments and coping with uncertainty led by motion blur. In general, although the VINS-Mono algorithm can roughly follow the real trajectory, it still has a large deviation in the local area, while our method has a more obvious inhibition of drift and a higher degree of agreement with the true value, which intuitively reflects the advantage of the adaptive covariance matrix in correcting errors in fast motion scenarios. The variation in each axial error and the global error with time is shown in the figures below.

**Figure 12:**
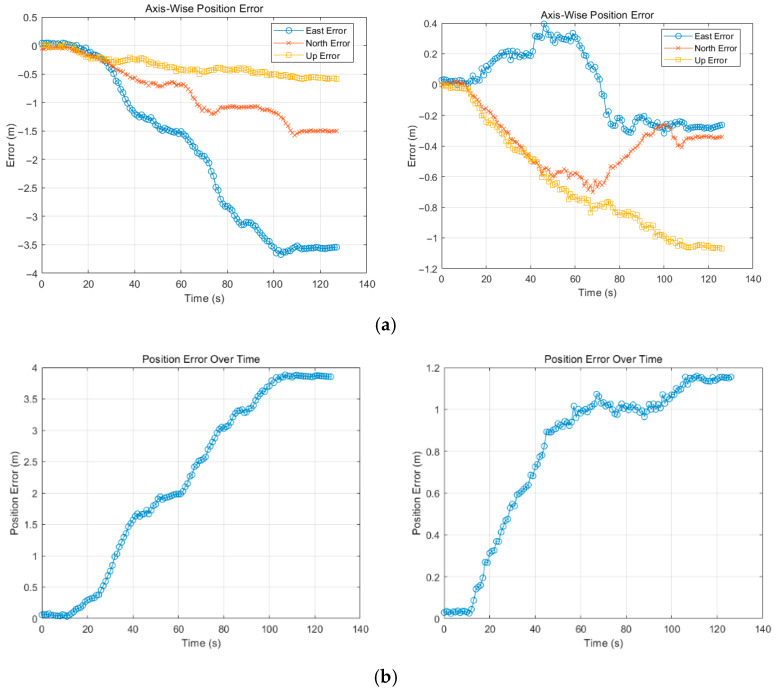
Results on F1 dataset (left: VINS-Mono, right: ours): (**a**) axial error, (**b**) global position error.

**Figure 13:**
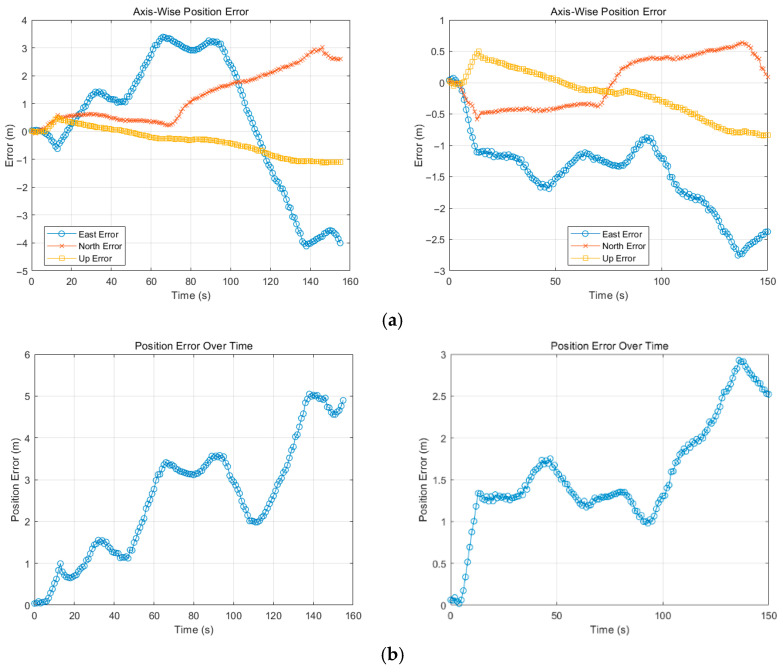
Results on F2 dataset (**left**: VINS-Mono, **right**: ours): (**a**) axial error, (**b**) global position error.

Figure 12 and Figure 13 illustrate the variation in the axial error relative to the RTK truth over time in two scenarios. As can be seen from the graph above, the errors in all three axes increase with time. Though our method performs slightly worse than VINS-Mono on the up direction in the F1 dataset, the error on the other two directions is kept under 1 m compared to the large deviation of VINS-Mono with north up to 1.5 m and east 3.5 m at the end. The gap can be clearly seen from the global position error in Figure 12b. As for the F2 dataset, due to the sparse features distributed within the surroundings, VINS-Mono cannot maintain enough precision when compared with our method, especially in the north direction. Even though the two methods follow a similar error growth trend in Figure 13a, our method diminishes the amplitude from 4 m to 2.7 m in the east, 3 m to 0.5 m in the north, and 1.1 m to 0.8 m in the up directions, showing great performance improvements.

Table 3 and Table 4 compare the quantitative positioning accuracy performance of VINS-Mono and ours in different time periods and directions, including RMS, STD, and MAE (median absolute error). By comparing various indicators, the positioning accuracy and stability of the two algorithms can be more intuitively evaluated in a short and long periods of time. In terms of error statistics in the first 60 s, our method is significantly better than the VINS-Mono in the eastward error and northward error. While the VINS-Mono is slightly better in height error, ours is still significantly better in terms of overall position error. With the extension of time, the cumulative drift of VINS-Mono begins to increase, resulting in a more obvious increase in RMS and STD. However, for Table 4, in the first 30 s, our error is slightly larger than that of the VINS-Mono, mainly because the system cannot accurately capture all the factors that affect image quality. The susceptibility of the Laplacian operator to noise can lead to noise being misinterpreted as a feature, leading to biased quality assessment. In the process of covariance matrix adjustment, if the influence of image blur is excessively amplified, the system may assign too much uncertainty to all observations and reduce the utilization rate of effective observation data. Conversely, reducing the covariance too much can lead to excessive trust in unreliable data and increase the risk of negative optimization. From the perspective of global error, ours has a better suppression effect on drift in long-term motion, and the optimization rate of RMS reaches 65.66% for the F1 dataset and 41.74% for F2, which verifies the effectiveness and robustness of the adaptive covariance matrix algorithm in complex environments or long-term operation.

## 4. Conclusions

In this study, we developed an innovative adaptive covariance matrix estimation method based on image quality assessment using Gaussian formulas to enhance the accuracy and robustness of UAV-based VINS. By dynamically adjusting the covariance matrix according to the assessed image quality, our method addresses the limitations of traditional VINS-Mono, which rely on fixed covariance settings and struggle under varying image conditions such as motion blur—a common challenge in UAV operations. Our approach leverages the advanced Laplacian operator to accurately assess image blur. Based on the results of these assessments, the system dynamically adjusts the visual covariance matrix, allowing it to adapt weight distribution according to the current image clarity. Extensive simulation experiments on the EuRoC and TUM VI datasets validated our method, demonstrating significant improvements in drone navigation accuracy under conditions with motion blur. Specifically, the proposed method consistently outperformed the VINS-Mono framework with performance improvements ranging from 3.87% to 52.56% (average 18.18%) across various sequences, as well as the optimization rate of RMS reaches 65.66% for the F1 dataset and 41.74% for F2 in the field tests outdoors. These findings further demonstrate the effectiveness of our adaptive covariance matrix estimation method in addressing the challenges faced by UAVs in dynamic environments. However, future works should concentrate on integrating visual, inertial, and additional sensor data (e.g., LiDAR or radar) to counteract image quality degradation besides motion blur.

## Figures and Tables

**Table 1:** Summary table of dataset.

Sequence	Environment	Key Features
MH01	Machine Hall	High-speed flight, cluttered environment
MH02	Machine Hall	Complex, unstructured environment, varying lighting
MH03	Machine Hall	Fast motion, dark scenes, high angular velocities
MH04	Machine Hall	Challenging navigation with dynamic obstacles
MH05	Machine Hall	High-speed, complex trajectories, low-light conditions
V101	Vicon Room	Controlled environment, standard trajectories
V102	Vicon Room	Varying viewpoints, moderate motion
V103	Vicon Room	Detailed feature tracking, controlled lighting
V201	Vicon Room	More complex paths, higher speeds
V202	Vicon Room	Diverse scene changes, increased difficulty
V203	Vicon Room	Long trajectories, mixed motion scenarios
Room1	Indoor Room	Moderate lighting, structured scenes with furniture
Room2	Indoor Room	Cluttered scenes, varied illumination
Room3	Indoor Room	Rapid motion, dynamic lighting changes

**Table 2:** Statistical table of optimization rate.

Dataset	Ours	VINS-Mono	Optimization Rate
MH01	0.226439	0.249729	9.33%
MH02	0.152096	0.160133	5.02%
MH03	0.292502	0.356147	17.87%
MH04	0.285233	0.367638	22.41%
MH05	0.297453	0.327509	9.18%
V101	0.18902	0.196633	3.87%
V102	0.183044	0.241726	24.28%
V103	0.283535	0.304758	6.96%
V201	0.134362	0.166646	19.37%
V202	0.202847	0.26214	23.23%
V203	0.325277	0.441438	26.31%
Room1	0.188013	0.224466	16.24%
Room2	0.266701	0.32504	17.95%
Room3	1.031089	2.17341	52.56%

**Table 3:** RMS, STD and MEAN trajectory errors on F1 dataset where bold marks the better result.

	Method	E (m)	N (m)	U (m)	3D (m)
RMS (60 s *)	VINS-Mono	1.03	0.58	0.23	1.20
	Ours	**0.22**	**0.41**	**0.41**	**0.62**
RMS (100 s)	VINS-Mono	1.91	0.89	**0.32**	2.13
	Ours	**0.22**	**0.48**	0.61	**0.81**
RMS (full)	VINS-Mono	2.32	1.06	**0.38**	2.58
	Ours	**0.23**	**0.46**	0.72	**0.88**
STD (60 s)	VINS-Mono	0.71	0.36	0.12	0.77
	Ours	**0.12**	**0.23**	0.26	0.35
STD (100 s)	VINS-Mono	1.15	0.48	**0.14**	1.22
	Ours	**0.21**	**0.22**	0.31	**0.37**
STD (full)	VINS-Mono	1.30	0.53	**0.17**	1.38
	Ours	**0.23**	**0.20**	0.35	**0.37**
MAE (60 s)	VINS-Mono	0.69	0.33	**0.22**	0.81
	Ours	**0.17**	**0.31**	0.35	**0.50**
MAE (100 s)	VINS-Mono	1.49	0.61	**0.32**	1.66
	Ours	**0.19**	**0.38**	0.55	**0.71**
MAE (full)	VINS-Mono	1.92	0.77	**0.37**	2.12
	Ours	**0.21**	**0.37**	0.65	**0.79**

* means under 60 s.

**Table 4:** RMS, STD, and MEAN trajectory errors on F2 dataset.

	Method	E (m)	N (m)	U (m)	3D (m)
RMS (60 s *)	VINS-Mono	**0.53**	0.46	**0.28**	**0.75**
	Ours	0.93	**0.39**	0.29	1.05
RMS (100 s)	VINS-Mono	2.04	0.63	0.24	2.15
	Ours	**1.21**	**0.37**	**0.20**	**1.28**
RMS (full)	VINS-Mono	2.39	1.53	0.58	2.90
	Ours	**1.58**	**0.41**	**0.42**	**1.69**
STD (60 s)	VINS-Mono	0.51	0.24	**0.16**	**0.41**
	Ours	**0.49**	**0.19**	0.17	0.52
STD (100 s)	VINS-Mono	1.29	0.33	0.24	1.15
	Ours	**0.41**	**0.25**	**0.19**	**0.40**
STD (full)	VINS-Mono	2.35	0.91	0.47	1.40
	Ours	**0.62**	**0.41**	**0.37**	**0.65**
MEAN (60 s)	VINS-Mono	**0.38**	0.40	**0.23**	**0.63**
	Ours	0.80	**0.34**	0.25	0.91
MEAN (100 s)	VINS-Mono	1.67	0.54	0.20	1.82
	Ours	**1.15**	**0.34**	**0.17**	**1.22**
MEAN (full)	VINS-Mono	2.02	1.24	0.45	2.54
	Ours	**1.46**	**0.38**	**0.33**	**1.56**

* means under 60 s.

## Data Availability

Data can be requested from corresponding author.
